# Antioxidant, Antithrombotic and Anti-Inflammatory Properties of Amphiphilic Bioactives from Water Kefir Grains and Its Apple Pomace-Based Fermented Beverage

**DOI:** 10.3390/antiox14020164

**Published:** 2025-01-29

**Authors:** Dimitra Papadopoulou, Vasiliki Chrysikopoulou, Aikaterini Rampaouni, Christos Plakidis, Anna Ofrydopoulou, Katie Shiels, Sushanta Kumar Saha, Alexandros Tsoupras

**Affiliations:** 1 Hephaestus, Laboratory, School of Chemistry, Faculty of Sciences, Democritus University of Thrace, St Lukas, 65404 Kavala, Greece; dipapnd@chem.duth.gr (D.P.); vgchrys@chem.duth.gr (V.C.); airabao@chem.duth.gr (A.R.); chrplak@chem.duth.gr (C.P.); anofrid@chem.ihu.gr (A.O.); 2 Centre for Applied Bioscience Research, Technological University of the Shannon: Midlands Midwest, Moylish Park, V94 E8YF Limerick, Ireland; katie.shiels@tus.ie (K.S.); sushanta.saha@tus.ie (S.K.S.)

**Keywords:** water kefir grains, water kefir beverage, bioactives, apple pomace, antioxidant, anti-inflammatory, antithrombotic

## Abstract

Kefir-based fermentation products exhibit antioxidant and anti-inflammatory effects against oxidative stress, inflammation, platelet activation and aggregation, and other related manifestations, thereby preventing the onset and development of several chronic diseases. Specifically, water kefir, a symbiotic culture of various microorganisms used for the production of several bio-functional fermented products, has been proposed for its health-promoting properties. Thus, water kefir grains and its apple pomace-based fermentation beverage were studied for bioactive amphiphilic and lipophilic lipid compounds with antioxidant, antithrombotic, and anti-inflammatory properties. Total lipids (TL) were extracted and further separated into their total amphiphilic (TAC) and total lipophilic content (TLC), in which the total phenolic and carotenoid contents (TPC and TCC, respectively) and the fatty acid content of the polar lipids (PL) were quantified, while the antioxidant activity of both TAC and TLC were assessed in vitro, by the ABTS, DPPH, and FRAP bioassays, along with the anti-inflammatory and antithrombotic activity of TAC against human platelet aggregation induced by the thrombo-inflammatory mediator, platelet-activating factor (PAF) or standard platelet agonists like ADP.ATR-FTIR spectra facilitated the detection of specific structural, functional groups of phenolic, flavonoid, and carotenoid antioxidants, while LC−MS analysis revealed the presence of specific anti-inflammatory and antithrombotic PL bioactives bearing unsaturated fatty acids in their structures, with favorable omega-6 (*n*-6)/omega-3 (*n*-3)polyunsaturated fatty acids (PUFA), which further support the findings that the most potent antioxidant, anti-inflammatory and antithrombotic bioactivities were observed in the TAC extracts, in both water kefir grains and beverage cases. The detection of such bioactive components in both the uncultured water kefir grains and in the cultured beverage further supports the contribution of water kefir microorganisms to the bioactivity and the bio-functionality of the final fermented product. Nevertheless, the extracts of the beverage showed much stronger antioxidant, anti-inflammatory, and antithrombotic activities, which further suggests that during the culture process for producing this beverage, not only was the presence of bioactive compounds produced by kefir microflora present, but biochemical alterations during fermentation of bioactive components derived from apple pomace also seemed to have taken place, contributing to the higher bio-functionality observed in the apple pomace—water kefir-based beverage, even when compared to the unfermented apple pomace. The overall findings support further studies on the use of water kefir and/or apple pomace as viable sources of antioxidant, anti-inflammatory, and antithrombotic amphiphilic bioactive compounds for the production of novel health-promoting bio-functional fermented products.

## 1. Introduction

According to the World Health Organization (WHO), non-communicable diseases such as cardiovascular diseases, cancer, chronic respiratory diseases, and diabetes are responsible for the majority of deaths worldwide each year [1]. Many chronic disorders, including those indicated above, have been associated with thrombo-inflammatory conditions and oxidative stress. More specifically, reactive oxygen species (ROS), thrombo-inflammatory mediators like platelet-activating factor (PAF), and classic platelet agonists like adenosine diphosphate (ADP) are all frequently involved in the onset of such inflammatory chronic diseases, which include increased platelet and leukocyte activation and aggregation, followed by oxidative damage and endothelial dysfunction at inflamed sites [2]. Various therapies that have been developed based on drugs acting against these pathways have been shown to be effective for the treatment and/or secondary prevention of such chronic diseases [2].

Primary prevention of such inflammatory chronic diseases can be achieved through the adoption of healthy eating habits [2]. Indeed, dietary bioactive compounds recovered from several sustainable healthy sources, such as fruits, have shown potent antioxidant and anti-inflammatory properties, protecting against oxidative and inflammatory processes that lead to associated pathologies [3,4]. At the same time, the by-products of various fruits have been shown to be suitable sources of phenolic compounds, polar lipids, and other antioxidant and anti-inflammatory bioactive components [3,5,6], which can be utilized for the development of health-promoting bio-functional products in a circular economy design [5].

Among these bio-functional products are those that are produced by fermentation processes. Fermented products, as well as the microorganisms involved in such fermentation processes, are of great interest since they possess various bioactive metabolites that act pleiotropically for health promotion [6,7,8,9]. Within this concept, many yeasts and other fermentative microorganisms can be used as sources of bioactive compounds for the development of functional foods, food supplements, cosmetics, or even drugs with health-beneficial properties such as antioxidant, anti-inflammatory, and antithrombotic effects [7,10].

One fermentation product that has gained popularity due to its demonstrated health-promoting properties is water kefir. Water kefir is a non-dairy beverage prepared by the fermentation of sugary solutions from kefir grains, which contain a mixture of probiotics consisting mainly of lactic acid bacteria, acetic acid bacteria, and yeast [11]. Several in vitro and in vivo studies have demonstrated the ability of both the individual microorganisms of water kefir and their bioactive metabolites and the total culture of water kefir and its fermentation products to promote health, exhibiting, among other effects, potent antioxidant, anti-inflammatory, and antithrombotic activities [12].

Apple pomace is a sugary substrate that can be used for the preparation of water kefir products [13]. Apple pomace, which is produced as a by-product during the processing of apples into apple juice and other apple-based products, is one of the most produced types of agri-food waste in the world. More specifically, the worldwide apple industry produces 70 million tons of apple pomace annually, which, due to its high content of bioactive compounds, could be exploited for the development of new functional products/foods with enhanced health-promoting properties, such as strong antioxidant, anti-inflammatory, and antithrombotic activities [5,6]. In this context, using apple pomace as a fermentation substrate for the preparation of water kefir could further enhance the bioactivity of the fermented product.

Thus, in the present study, amphiphilic and lipophilic lipid extracts of water kefir grains (WKGs) and an apple pomace-based water kefir beverage (WKB) were evaluated for their total carotenoid and phenolic contents, as well as for their potential antioxidant, antiplatelet, and anti-inflammatory properties. At the same time, the ATR-FTIR spectra of the amphiphilic extracts of the samples, as well as the estimation of their fatty acid composition by LC−MS analysis, facilitated a correlation between the structure and activity of the bioactive components in these extracts, which exhibited the highest bioactivity.

## 2. Materials and Methods

### 2.1. Materials, Reagents and Instrumentation

Water kefir grains were purchased from The Ferment Company (Hillegom, The Netherlands) [14]. Apple pomace was produced during the preparation of apple juice from organic-cultured apples of the Starking variety in Zagora, Greece.

All reagents, including Folin-Ciocalteu, 1,1-diphenyl-2-picrylhydrazyl (DPPH) and 2,2′-azinobis-(3-ethylbenzothiazoline-6-sulfonic acid (ABTS), solvents (chloroform, methanol, petroleum ether, ethanol, n-octane, isopropanol), standard phenolics (trolox, gallic acid, quercetin, catechin) and lipid standards (soybean polar lipids, β-carotene) were purchased from Sigma Aldrich (St. Louis, MO, USA). UV-Vis spectroscopy analyses were performed using an LLG-uniSPEC 2 spectrophotometer, and ATR-FTIR spectroscopy was performed using a Perkin Elmer Frontier ATR/FT-NIR/MIR spectrometer.

All plastic consumables, reagents, and solvents used in the antiplatelet assays were of analytical grade and were purchased from Sigma Aldrich. 20 gauge (G) safety needles and evacuated sodium citrate S-monovettes^®^ for blood sampling were purchased from Sarstedt Ltd. (Wexford, Ireland). Bioassays on human PRP (hPRP) were performed using a Chrono-log-490 (Havertown, PA, USA) quatriple channel strobilometric platelet aggregator connected to the accompanying AGGRO/LINK software package. All consumables used for platelet aggregation were purchased from Chrono-log (Havertown, PA, USA). Platelet-activating factor (PAF), ADP, and bovine serum albumin (BSA) standards were purchased from Sigma Aldrich (St. Louis, MO, USA). Centrifugation was performed using a 4000 rpm maximum capacity Nahita Blue Medibas centrifuge.

### 2.2. Preparation of WKGs and WKB Samples

The present experiment was carried out with triplicate samples to ensure the repeatability of the results. The apple juice (desiccated) and the remaining by-products in the form of pomace were obtained separately from the apples using a juicer.

Regarding the samples of water kefir grains, each of them was formed from a concentration of 10 g of commercial water kefir grains. To prepare the water kefir beverage, 1 L of sterilized boiling water (boiling time = 10 min) and one spoonful (18 g) of granulated sugar were initially transferred to each of the three beakers.

The resulting solution was stirred with a spoon and left for 10 min at a temperature of 66 °C (checked with a thermometer) to achieve pasteurization of the added solutions freshly produced from the juicer apple pomace material. More specifically, 100 g of the just-collected from the juicer apple pomace was transferred to each of the three solutions at 66 °C for 10 min (checked with a thermometer) to achieve pasteurization, and then all solutions were covered with a sterilized cotton cloth and placed at room temperature (20 °C) in a pre-sterilized lab-bench. The pH (pH = 7) was then checked using a pH paper. Finally, 5 g of water kefir grains was added to each solution, where they were activated in the presence of sugary fermentation substrates (sugar, apple pomace). The fermentation of apple pomace was then carried out at 20 °C in an aerobic environment, which was stopped at 60 h when the pH of the solution was equal to 5, avoiding further fermentation that would lead to the production of apple cider vinegar.

### 2.3. Extraction of Lipid Bioactives from WKGs and WKB and Separation of Their Total Amphiphilic (TAC) and Total Lipophilic Content (TLC)

Total lipids (TLs) of three samples of both water kefir grains (10 g/sample) and water kefir beverage (40 mL/sample) were extracted using a modified version of Bligh and Dyer’s method [15], according to Tsoupras et al. [5]. The TLs obtained were further separated into their total lipophilic content (TLC) and total amphiphilic content (TAC) by counter-current distribution, based on a modification of the methodology of Galanos and Kapoulas [16], as described in previous studies [5,17]. More specifically, extraction of TLs was performed by homogenizing each sample in a single-phase system containing chloroform/methanol/water at a ratio of 1:2:0.8 (*v*/*v*/*v*). In the case of the water kefir grain samples, a blender was used to homogenize the solid grains. For extraction, each WKG sample was blended for 2 min, and after being left at rest for 1 min, it was blended for an additional 2 min. The extraction of TLs of the samples of water kefir beverages in the presence of apple pomace was carried out immediately after the preparation of these beverages. The homogeneous solution for each sample that is at a ratio of 1:2:0.8 (*v*/*v*/*v*) was moved to a separation funnel, to which chloroform and water were added in the right proportions to adjust the homogenate to a chloroform/methanol/water ratio of 1:1:0.9 (*v*/*v*/*v*) to achieve phase separation. TLs extracted from the samples that were in the chloroform layer were gathered in spherical flasks, and the solvents (primarily chloroform) were evaporated in a rotary evaporator under vacuum. This was followed by the collection of the remaining TLs from the spherical flasks by redissolving them in small volumes (1–3 mL) of 1/1 (*v*/*v*) chloroform/methanol solution, which facilitated their transfer into small preweighed glass tubes.

Both TLC and TAC of TLs were obtained by counter-flow distribution using the pre-equilibration of petroleum ether for the collection of TLC extracts and 87% ethanol in water for the collection of TAC extracts. After separation, the solvents from the TLC and TAC extracts were re-evaporated under vacuum in a rotary evaporator before being moved to small, preweighed glass tubes using small volumes (1–3 mL) of petroleum ether for TLC extracts and 1/1 (*v*/*v*) chloroform/methanol solution for TAC extracts.

A nitrogen stream was used to evaporate the solvents used for transferring the extracted lipids into all tubes, and the obtained TL, TAC, and TLC extracts of each sample were weighed to obtain the yield of extraction and then stored at −20 °C for further analysis.

For all the following analyses, each of the dry TAC extracts was dissolved in 1 mL of ethanol, while each dry TLC extract was dissolved in 1 mL of octane, and all were equally aliquoted into several individual glass tubes, where all solvents were again evaporated under a nitrogen stream to obtain dry TAC and TLC samples for further analysis.

### 2.4. Total Carotenoid Content (TCC) Analysis

The total carotenoid content (TCC) of each extract was determined according to Tsoupras et al. [5]. Specifically, each sample was dissolved in 2 mL of octane, and its absorbance was measured at 450 nm. The concentration sought was measured based on the β-carotene standard curve, which was produced using several concentrations of β-carotene dissolved in octane between 1 and 50 μg/mL. Appropriate dilutions of the dissolved octane extract were also prepared in order to obtain absorbance values within the standard curve of β-carotene. The obtained results were expressed in mg β-carotene equivalent (CE)/g extract.

### 2.5. Total Phenolic Content (TPC) Analysis

The total phenolic content (TPC) of all extracts was evaluated using the Folin-Ciocalteu reagent, according to Tsoupras et al. [5], with some modifications. More specifically, 1 mL of distilled water and 1 mL of Folin-Ciocalteu reagent were added to each sample. After 7 min, 3 mL Na_2_CO_3_ was added to each sample. This was followed by incubation of the samples in the dark for 2 h. Between successive additions of reagents and every 30 min during incubation, the solutions were stirred using a vortex. After the two-hour incubation of the samples, their absorbance at 765 nm was measured. The concentration sought was measured based on the gallic acid standard curve, which was produced using several concentrations of gallic acid between 0.5–10 μg/mL. Appropriate dilutions of the extract were also prepared in order to obtain absorbance values within the standard curve of gallic acid. The results obtained were expressed in mg gallic acid equivalent (GAE)/g extract.

### 2.6. Assessment of Antioxidant Activities of Extracts

The evaluation of the antioxidant activity of the samples was carried out by three different assays, based on the DPPH radical scavenging assay, but also through the ABTS radical cation decolorization method, as well as by the ferric-reducing antioxidant power (FRAP) method, according to Tsoupras et al. [5] and Xiao et al. [18], with some modifications.

For the DPPH assay, 0.2 mL of ethanol, 0.8 mL of Tris-HCl buffer (pH 7.4), and 1 mL of DPPH solution were transferred to each sample. Between successive additions of the reagents, the solutions were stirred using a vortex. The solutions were left at room temperature for 30 min, and immediately afterwards, their absorbance was recorded at 517 nm. The percentage of inhibition (%) was calculated using the following equation:

Inhibition (%) = (A1 − A2) × 100/A1(1)

where A1 is the absorbance of the control sample solution and A2 is the absorbance of the test sample solution.

The IC50, i.e., the concentration of each extract that has the ability to neutralize 50% of the DPPH radical, was then calculated. The DPPH radical scavenging activity of the sample was expressed as the Trolox equivalent antioxidant capacity (TEAC). TEAC was calculated as follows:

TEAC = IC50 of Trolox (μg/L)/IC50 of the sample (μg/L)(2)

For the ABTS assay, 2 mL of ABTS solution was added to each sample, followed by vortex stirring. The solutions were incubated in the dark for 7 min, and immediately afterwards, their absorbance was measured at 734 nm. Trolox was used as the standard. The concentration of Trolox was chosen under the condition that the absorbance value ranged from 0.2 to 0.8, for drawing a standard curve.

The result was expressed as μmol TE/g DW according to the following formula:

ABTS (μmol TE/g DW) = c × V × t/m(3)

where c is the Trolox concentration (µmol/mL) of the corresponding standard curve of the diluted sample, V is the volume of the sample (mL), t is the dilution factor, and m is the dry weight of the sample (g).

For the FRAP assay, 3 mL of FRAP solution was transferred to each sample, followed by vortex stirring. The solutions were incubated in the dark at 37 °C for 15 min, and immediately afterwards, their absorbance was measured at 593 nm. Trolox was used as a standard. The concentration of Trolox was chosen under the condition that the absorbance value ranges from 0.2 to 0.8, for drawing a standard curve.

The result was expressed as μmol TE/g DW according to the following formula:

FRAP (μmol TE/g DW) = c × V × t/m(4)

where c is the Trolox concentration (µmol/mL) of the corresponding standard curve of the diluted sample, V is the volume of the sample (mL), t is the dilution factor, and m is the dry weight of the sample (g).

### 2.7. Assessment of Antiplatelet and Anti-Inflammatory Properties of Extracts with Light Transmittance Aggregometry

Bioassays for the evaluation of the antiplatelet and anti-inflammatory properties of all the extracts were carried out on human platelet-rich plasma (hPRP) preparations from healthy donors (*n* = 6), evaluating their ability to inhibit human platelet aggregation when hPRP was initiated through the inflammatory and thrombotic mediator PAF and via the well-established platelet agonist ADP, in the presence of these extracts, as previously described [4,5,17,19]. The efficacy of the anti-inflammatory and antithrombotic activities of all samples was expressed as the mean of their IC50 values (half maximum inhibitory concentration) ± standard deviation (SD), quantified in mass (μg) of the bioactive lipid extract present in the aggregometer cuvette that resulted in 50% inhibition of PAF- or ADP-induced hPRP aggregation, as previously described [4,5,17,19]. In order to ensure reproducibility, every sample was evaluated several times in blood samples from different healthy donors (*n* = 6).

### 2.8. ATR-FTIR Analysis

In order to obtain the spectra of the TAC extracts and of the five standards (β-carotene, gallic acid, catechin, quercetin, and polar lipids from soybean), we applied the ATR-FTIR spectrophotometer technique (Perkin Elmer Frontier ATR/FT-NIR/MIR spectrometer) at a wavenumber range of 4000–600 cm^−1^ with 32 scans, according to Vordos et al. [20]. Each sample analyzed was first dissolved in a small volume of isopropanol (1–3 mL), and then placed on the plate in an amount such that it covered the crystal. After that, the tip was adjusted to rest on the plate. Once these are in contact, a green line appears on the Force Gauge, and the force applied through the rotating tower is increased until the spectrum displayed on the work surface is stabilized. The spectrum obtained for each sample was analyzed according to the standard samples for which the same procedure was previously followed (Supplementary Figure S1). In parallel, the spectrum of isopropanol was also analyzed to reduce the error in the peak study due to solvent interference in the sample under study. All spectra were corrected against the air background spectrum.

### 2.9. LC–MS Analysis

Liquid chromatography-mass spectrometry (LC−MS) was used to elucidate the overall structures of the PLs in the TAC extracts and their fatty acid composition derived from their saponification, as described by Tsoupras et al. [5,19]. Briefly, each of these lipid samples was separated into two halves and dried under a N_2_ stream. The first half of each sample was saponified by adding 1.5 mL of a saponification reagent (2.5 M KOH: methanol (1:4, *v*/*v*)), which was gently vortexed. The vials were incubated at 72 °C for 15 min prior to the addition of 225 µL formic acid. Then, 1725 µL of chloroform and 375 µL of Milli-Q water were added and vortexed to separate the two layers. The chloroform layer containing FFA was transferred carefully to amber vials and evaporated to dryness before being stored at −20 °C until LC−MS analysis.

Before LC−MS analysis, re-dilution of each extract was performed in 500 μL dichloromethane/methanol (1:2, *v*/*v*), followed by centrifugation for 6 min at 13,000 r/min (Heraeus Biofuge Stratos, Fisher Scientific Ltd., Dublin, Ireland). Filtration of clear supernatants was carried out by the use of 3 kDa ultracentrifuge filters (Amicon Ultra 3 k, Merck Millipore Ltd., Darmstadt, Germany. Fatty acid profiles in these filtrates were obtained by injecting 10 μL of each filtrate into an HPLC system (Agilent 1260 series, Agilent Technologies Ireland Ltd., Little Island, Co., Cork, Ireland) equipped with a Q-TOF mass spectrometer (Agilent 6520) fitted with electrospray ionization (ESI) as the source type. For the separation of fatty acids, an Agilent C18 Poroshell 120 column (2.7 μm, 3.0 × 150 mm) with gradual elution was used, which had mobile phase A composed of 2 mM ammonium acetate in water and mobile phase B composed of 2 mM ammonium acetate in 95% acetonitrile. The mobile phase had an initial flow rate of 0.3 mL/min until 5 min elapsed and was increased to 0.6 mL/min after 10 min and then maintained at this flow rate until the end of the run. The mass spectrometer scanned *m*/*z* from 50 to 1100, while reference masses 1033.988 and 112.9855 were used for monitoring the scan in the negative ionization mode. The capillary voltage was 3500 V, and the scatterer and fragmenter voltages were maintained at 65 V and 175 V, respectively. The pressure, drying gas flow, and nebulizer temperature were set at 30 psi, 5 L/min, and 325 °C, respectively.

The LC−MS method was validated by the comparison of specific gravity and retention time (RT) of various standard saturated and unsaturated fatty acids, i.e., lauric (C12:0), myristic (C14:0), palmitic (C16: 0), stearate (C18:0), oleic (OA, C18: 1n-9 cis), linoleic (LA, C18:2 n−6 cis), gamma-linolenic (GLA, C18:3 *n*-6), α-linolenic (ALA, C18:3 *n*-3), arachidonic (ARA, C20: 4 *n*-6), eicosapentaenoic acid (EPA, 20:5 *n*-3), docosahexaenoic acid (DPA, 22:5 *n*-3) and docosahexaenoic acid (DHA, 22:6 *n*-3) (Sigma, Ireland). These standards further facilitated the assessment of each lipid extract sample, in which all of its fatty acids were identified based on their known exact mass. The average of the triplicate samples represented the peak area of each identified fatty acid, and their relative content was recorded based on their average peak area (Supplementary Figures S2 and S3). Considering that the peak areas do not reflect the exact proportions of individual fatty acids, a cautious reading of the relevant data is required.

The assignment of FFA and phospholipid species was based upon a combination of survey, daughter, precursor, and neutral loss scans, and the identity of the bioactive PL molecules was verified using the LIPID MAPS: Nature Lipidomics Gateway (www.lipidmaps.org, accessed on the 8 January 2025) by using the lowest delta values combined with the results obtained from the LC−MS analysis on the fatty acid composition of the saponified PL, as previously described by Tsoupras et al. [5,19].

### 2.10. StatisticalAnalysis

One-way analysis of variance (ANOVA) was used for all comparisons of IC_50_ values against PAF-induced aggregation of human platelets, while Kruskal−Wallis nonparametric multiple comparison tests were used for all other comparisons. Differences were considered statistically significant when the *p* value was less than 0.05 (*p* < 0.05). The data were analyzed using a statistical software package (IBM-SPSS statistics 26 for Windows, SPSS Inc., Chicago, IL, USA).

## 3. Results and Discussion

### 3.1. Yield Extraction of Lipids from WKGs and WKB

The extraction and separation of amphiphilic (TAC), lipophilic (TLC), and total (TL) lipid compounds from water kefir grains and water kefir beverage was based on the Bligh and Dyer extraction method [15] and the counter-current distribution technique of Galanos and Kapoulas [16], as described by Tsoupras and others [5]. This combination of methods has been applied in previous studies to efficiently recover and separate polar and neutral lipids from various plant sources, such as apple pomace, for further evaluation of their bioactive properties [17,21]. In fact, it has been reported that, unlike other extraction methods, this combined technique ensures the preservation of the lipid components’ bioactivity during the extraction process [22], which is verified by the high bioactivity that the tested extracts exhibited in the antioxidant and anti-inflammatory/antithrombotic activity assays.

Table 1 shows the extraction yields for the amphiphilic (TAC), lipophilic (TLC), and total (TL) lipids recovered from water kefir grains and water kefir beverage, expressed in g per 100 g of grains or g per 100 mL of beverage, respectively. As can be observed, higher amounts of amphiphilic than lipophilic lipid components were found in both water kefir grains and beverage extracts. The above result is significant, as it has been reported that unlike lipophilic and total lipids, which exhibit low and intermediate bioactivity, respectively, amphiphilic lipids are the most bioactive lipid class [17]. Additionally, by applying the method of Tsoupras et al. [5], which constitutes a modification of other lipid extraction techniques, satisfactory extraction yields, mainly of amphiphilic lipid components from water kefir grains, were achieved, which were even higher than other yields previously reported for the extraction of lipids from microorganisms used for the preparation of water kefir [23].

### 3.2. Total Carotenoid Content (TCC) of Extracts from WKGs and WKB

Table 2 shows the total carotenoid content of amphiphilic (TAC) and lipophilic (TLC) lipid extracts of water kefir grains and water kefir beverage, expressed in mg of β-carotene equivalents (CE) per g of extract. As shown, mainly the lipophilic (TLC) but also the amphiphilic (TAC) lipid extracts of both the water kefir grains and the water kefir beverage were rich in carotenoids (the max, median, and min values of carotenoid concentration in the amphiphilic extracts of each sample were found to be lower than those in its lipophilic extracts). Indeed, the presence of these bioactive compounds has been reported both in the lipophilic phase of food sources, such as milk, where they constitute part of its lipid-soluble antioxidant components [24] and in the polar lipid extracts of natural sources, such as in apple pomace amphiphilic lipid extracts [5].

The total carotenoid content of kefir grains was investigated for the first time in the present study. As shown in Table 2, the concentration of carotenoids in the water kefir grain extracts was lower than that found in the extracts of the fermented beverage (the max, median and min values of carotenoid concentration in the WKB extracts were found to be higher than those in WKGs extracts). This result can be attributed to the fact that these bioactive compounds detected in the fermented product are mainly produced by the water kefir microbiota during the fermentation process [25]. However, the appreciable amount of carotenoids found in the extracts of unactivated/uncultivated water kefir grains suggests the presence of these bioactive components in the cells of water kefir microorganisms and, therefore, the contribution of these microorganisms to the bioactivity of the final product.

Other recent studies have also established the high carotenoid content of various probiotic water kefir beverages [26,27,28]. In one of them, the enrichment of a soy milk-based water kefir beverage with by-products from the plant *Acrocomia aculeata* resulted in increased levels of carotenoids, which was attributed to the high content of these bioactive compounds in this plant [28]. Indeed, carotenoids occur naturally in various fruits and vegetables [29]. Thus, the high amount of carotenoids detected in the extracts of the water kefir beverage could also be related to the use of apple pomace as a fermentation substrate. More specifically, apple pomace is rich in carotenoids, with lutein and β-carotene being the most abundant ones [30]. In fact, extracts of apple pomace products, which have been found to contain, among other bioactive compounds, carotenoids with strong antioxidant, antithrombotic, and anti-inflammatory properties, have been used as bioactive constituents for the production of biofunctional foods such as bakery [5] and dairy [31] products.

### 3.3. Total Phenolic Content (TPC) of Extracts from WKGs and WKB

Table 3 shows the total phenolic content of amphiphilic (TAC), lipophilic (TLC), and total lipid (TL) extracts of water kefir grains and water kefir beverage, expressed in mg of gallic acid equivalents (GAE) per g of extract. As shown, mainly the amphiphilic (TAC) and the lipophilic (TLC) lipid extracts of both the water kefir grains and the water kefir beverage were rich in phenolic compounds (the max values of phenolic concentration in the amphiphilic extracts of each sample were found to be higher than those in its lipophilic extracts). Bioactive amphiphilic phenolic compounds have also been isolated from other natural sources, such as apple pomace [17].

The total phenolic content analysis of unactivated/uncultivated water kefir grains was carried out for the first time in the present study. As shown in Table 3, the total phenolic content of the extracts of the water kefir beverage was higher than that of the extracts of the grains (the max, median and min values of phenolic concentration in the WKB extracts were mostly found to be higher than those in WKGs extracts). This finding is in agreement with the results of previous studies that have demonstrated a significant enhancement of phenolic content and, consequently, of the bioactivity of other fermented beverages due to the release of phenolic components during the breakdown of complex phenolic compounds by the enzymes produced by the microorganisms of water kefir [32,33]. More specifically, bacteria and yeasts naturally present in water kefir grains produce enzymes capable of breaking down complex structures, including those formed by the association of phenolic compounds with proteins or carbohydrates, releasing free phenolic compounds from the fermentation substrate or kefir grains [34,35]. In the case of our beverage, these enzymes could break down the complexes formed by the phenolic compounds of apple pomace with carbohydrates, lignin, pectin and proteins, leading to the diffusion of free phenolic compounds with increased bioactivity [36,37]. However, along with their metabolites, the microorganisms themselves contribute to the bioactivity of the fermented product, which is confirmed by the significant amount of phenolic compounds found in the extracts of unactivated water kefir grains.

Fermentation with water kefir grains has emerged as an efficient method for enhancing the total phenolic content of a product [38]. Various types of plant-based proteins have shown increased total phenolic content after their fermentation with water kefir grains [34,39,40]. At the same time, various beverages fermented with water kefir or with individual strains of its microbiota have shown a high concentration of total phenolic compounds and, consequently, strong antioxidant activities [41,42,43,44], as well as other health-promoting properties such as anti-apoptotic and neuroprotective effects [45]. Indeed, in a study similar to the present one, the use of apple pomace as a fermentation substrate led to an increase in the antioxidant capacity of a pitaya-based water kefir beverage, which was attributed to the high phenolic content of apple pomace [13]. Indeed, apple pomace is rich in phenolic substances, which contribute directly to its antioxidant activity [5,6,46]. According to the literature, these phenolic compounds are mainly composed of phenolic acids (such as 3,4-dihydroxybenzoic acid, gallic acid, 4-hydroxybenzoic acid, and chlorogenic acid), flavonoids (such as florizin, epicatechin, catechins, flavonol, quercetin, and dihydrochalcones), and anthocyanins [47,48,49]. The content of phenolic compounds in apple pomace depends on the variety of apples, growing conditions, and processing parameters during juice extraction, which can affect the extractability of certain phenolic compounds from fruit tissues [50]. Extracts from apple pomace by-products have been used several times for the preparation of various nutritional products with high total phenolic content and, consequently, strong antioxidant capacity [5,47,51].

### 3.4. Antioxidant Activity of Extracts from WKGs and WKB

The results of the antioxidant assay methods, ABTS, DPPH, and FRAP, for all examined extracts are presented in Table 4. In all three assays, only the amphiphilic (TAC) lipid extracts of the water kefir grains and beverage showed strong antioxidant activity, which seems to be consistent with the higher total phenolic content (TPC) of these extracts compared to that found for the lipophilic (TLC) lipid extracts of the samples, which showed no or low bioactivity in these antioxidant activity tests. Therefore, the most bioactive compounds were mainly present in the amphiphilic lipid extracts of water kefir. These results are probably related to the different lipophilicities of TAC and TLC lipid extracts, as the more hydrophilic phenolic bioactive components of TAC seem to be more effective in neutralizing free radicals and reducing trivalent iron than lipophilic carotenoids. The high ABTS, TEAC, and FRAP values highlight the ability of these bioactive components to act against oxidative stress and related inflammatory events and disorders through various antioxidant mechanisms, including free radical scavenging and high-valent metal reduction.

As shown in Table 4, the antioxidant activity exhibited by the water kefir beverage extracts in the ABTS, DPPH, and FRAP assays was statistically significantly stronger than that of the grain extracts. The above result could be related to the higher phenolic and carotenoid contents of the beverage extracts, as determined by TPC and TCC analysis, respectively. Similarly, previous studies have also reported an association between the antioxidant activity of kefir beverages estimated by these methods and their high levels of phenolic compounds and carotenoids [24,44]. The observed antioxidant capacity of water kefir products is mainly attributed to the action of bioactive metabolites produced by microorganisms in water kefir during fermentation. These metabolites include compounds with direct antioxidant activities, such as exopolysaccharides (EPSs) produced by lactic (LAB) and acetate (AAB) bacteria [52]. At the same time, β-glucosidase, an enzyme also produced by the microflora of water kefir, has the ability to hydrolyze the glycosidic forms of isoflavones present in the beverage and convert them to the corresponding non-glycosidic forms, which have a stronger antioxidant activity, thus improving the antioxidant capacity of the final product [53].

Furthermore, the antioxidant capacity of water kefir products is significantly enhanced by the action of bioactive compounds present in the fermentation substrate [54]. In a related study, it was found that some of the antioxidants present in apple pomace, which was used as a fermentation substrate, were retained in the fermented beverage [13]. Apple pomace is rich in antioxidants, including mainly polyphenols, vitamins, and pectin [55]. The polyphenolic compounds in apple pomace are primarily flavonoids (such as quercetin and phloretin), followed by phenolic acids (such as caffeic acid, chlorogenic acid, and catechin) [56]. The majority of polyphenols are found in their bound/glycosidic forms, which exhibit lower antioxidant capacities than their aglycones. However, the fermentation of apple pomace by lactic acid bacteria (LAB) with high β-glucosidase activity, such as those present in water kefir grains, can lead to the conversion of the contained polyphenol glycosides to their corresponding non-glycosidic forms, enhancing the antioxidant activity of the pomace [57].

### 3.5. Anti-Inflammatory and Antiplatelet Properties of Extracts from WKGs and WKB

**Figure 1:**
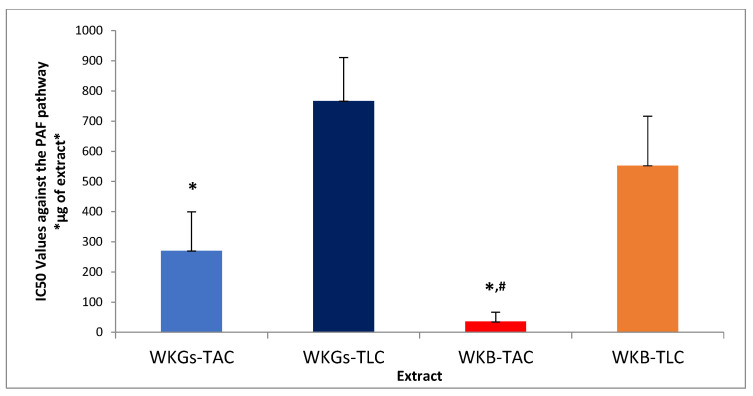
The anti-inflammatory and antithrombotic potency of extracts from WKGs and WKB against human platelet aggregation induced via the inflammatory and thrombotic mediator PAF. Results are expressed as means of the IC50 (half-maximal inhibitory concentration) values in μg of TAC and TLC extract in the aggregometer cuvette that causes 50% inhibition of PAF-induced platelet aggregation (the lower the IC50 value for a lipid extract, the higher its inhibitory effect against the specific agonist of platelet aggregation). * denotes a statistically significant difference, *p* < 0.05, of the anti-PAF anti-inflammatory activity of TAC extracts compared to TLC extracts in both kefir grains and kefir beverage, while # denotes a statistically significant difference, *p* < 0.05, of the anti-PAF activity of beverage TAC extracts compared to kefir grains TAC extracts. Abbreviations: WKGs-TAC, amphiphilic lipids extracted from water kefir grains; WKGs-TLC, lipophilic lipids extracted from water kefir grains; WKB-TAC, amphiphilic lipids extracted from water kefir beverage; WKB-TLC, lipophilic lipids extracted from water kefir beverage.

**Figure 2:**
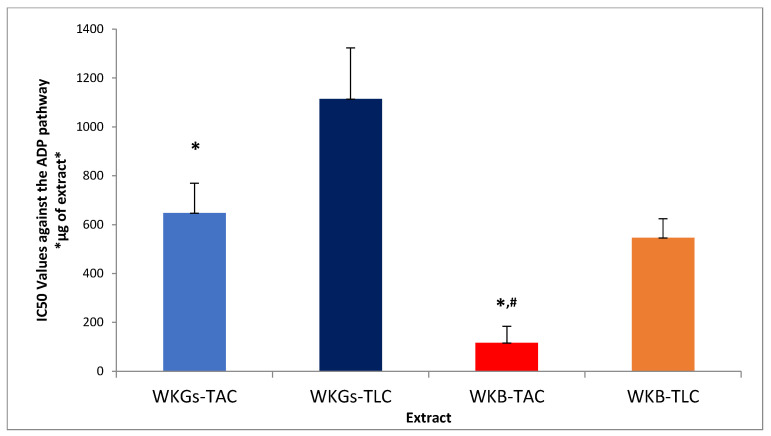
The antiplatelet potency of extracts from WKGs and WKB against human platelet aggregation induced by a standard platelet agonist, ADP. Results are expressed as means of the IC50 (half-maximal inhibitory concentration) values in μg of TAC and TLC extract in the aggregometer cuvette that causes 50% inhibition of ADP-induced platelet aggregation (the lower the IC50 value for a lipid extract, the higher its inhibitory effect against the specific agonist of platelet aggregation). * denotes a statistically significant difference, *p* < 0.05, of the anti-ADP antiplatelet activity of TAC extracts compared to TLC extracts in both kefir grains and kefir beverage, while # denotes a statistically significant difference, *p* < 0.05, of the anti-ADP activity of beverage TAC extracts compared to kefir grain TAC extracts. Abbreviations: WKGs-TAC, amphiphilic lipids extracted from water kefir grains; WKGs-TLC, lipophilic lipids extracted from water kefir grains; WKB-TAC, amphiphilic lipids extracted from water kefir beverage; WKB-TLC, lipophilic lipids extracted from water kefir beverage.

The anti-inflammatory and antiplatelet efficacy (IC50 value) of amphiphilic (TAC) and lipophilic (TLC) lipid extracts of water kefir grains and beverage against human platelet aggregation caused by the inflammatory and thrombotic mediator PAF or by a classical platelet agonist (ADP) are summarized in Figure 1 and Figure 2, respectively. As it is shown, all the tested extracts exhibited inhibitory activity against both PAF-induced and ADP-induced human platelet aggregation. PAF is one of the best-known mediators of both inflammation and thrombosis [2], with ADP also being a potent thrombotic agent on platelets acting through the arachidonic acid pathway [2]. By binding to their specific receptors, which are located on the surface of various cells, including platelets, these agonists activate proinflammatory processes through agonist-specific signaling pathways, leading to the pathogenesis of thrombo-inflammation related chronic diseases [2]. The platelet aggregation model used in the present study is an ideal tool to reveal the substances and their quantities that are capable of attenuating the action of these thrombo-inflammatory factors and, consequently, reducing the risk of diseases related to the activation of platelets and the signaling pathways of their agonists, such as cardiovascular diseases (CVDs) and cancer [2]. The potent inhibitory activity exhibited by the tested extracts in these assays demonstrates the potential application of water kefir and its products as sources of bioactive constituents to combat thrombo-inflammatory processes and prevent related diseases.

According to Figure 1 and Figure 2, the amphiphilic (TAC) lipid extracts of both the water kefir grains and the water kefir beverage had lower IC50 values than their lipophilic (TLC) lipid extracts in the two cases of agonists. Therefore, a lower concentration of amphiphilic rather than lipophilic lipid components is required to induce 50% inhibition of platelet activation, demonstrating stronger antiplatelet activity. The latter seems to be consistent with the higher content of total phenolic components (TPC) and polar lipids in TAC extracts compared to that calculated for the TLC extracts of the samples, which showed the lowest antiplatelet effects (higher IC50 values). These findings are in agreement with the results of the antioxidant activity assays and suggest stronger anti-inflammatory and antithrombotic effects of the amphiphilic phenolic compounds and polar lipids in the TAC extracts compared to the corresponding effects of the lipophilic carotenoids in the TLC extracts.

As shown in Figure 1 and Figure 2, the extracts of the water kefir beverage showed stronger antiplatelet activity (lower IC50 values) than the extracts of the water kefir grains, both in the case of platelet activation by PAF and in the platelet aggregation induction assay by ADP. The anti-inflammatory activity of water kefir fermentation products, which has also been reported by other researchers, can be attributed to the presence of its microorganisms and the various bioactive compounds they produce during fermentation [58,59]. In related studies, polar lipids extracted from various microorganisms found in water kefir grains, as well as from their fermentation products, have demonstrated strong inhibitory effects against PAF and ADP signaling pathways [9,60]. In the case of our beverage, these effects were attributed to the presence of apple pomace bioactive compounds in the final product, such as polar lipids, phenolic compounds, and carotenoids, which have been reported to have anti-inflammatory and antithrombotic abilities [5]. Interestingly, the inhibitory activity of the extracts of the amphiphilic lipid components of the beverage prepared by fermentation of apple pomace with water kefir against PAF and ADP was stronger than that reported for the extracts of the amphiphilic lipid components of unfermented apple pomace [5]. This demonstrates the importance of the fermentation process in enhancing the bioactivity of fermented raw materials.

By comparing the IC50 values of each tested extract against two different platelet-activating factors, we observed that all extracts showed significantly stronger inhibitory activity in the assay of platelet activation with PAF than with ADP. The exception was the extract of the lipophilic lipid components of the water kefir beverage, which showed almost equal antiplatelet activity in both platelet aggregation assays. The observed differences in the inhibitory activity of the bioactive components of each extract against the signaling pathways of the two different activating factors could be attributed to the different structures of their receptors and/or to the different ways in which these bioactive compounds bind to the receptors of the two agonists. In general, the bioactive components of our samples seem to be more active and specific against the PAF signaling pathway, probably due to their inhibitory activity against PAF binding to its receptor. This is mostly because of the presence of bioactive polar lipids containing unsaturated fatty acids, which have been found to have a strong antagonistic effect against PAF binding to its receptor due to their structural similarity to PAF and, thus, their relative chemical affinity for the PAF receptor. In addition, they can release unsaturated fatty acids (after membrane phospholipase A2 action), which intracellularly inhibit the inflammatory eicosanoid cycle while also promoting the production of resolvins that stimulate the termination of the inflammatory response [2]. However, further research is required to elucidate the mechanism of action of the bioactive components of water kefir and its products, and how this relates to each platelet-activating factor. In any case, the strong inhibitory activity exhibited by the extracts of the water kefir grains and beverage against the PAF signaling pathway is very important since, in contrast to ADP, whose role is mainly limited to platelet activation and aggregation, PAF is a powerful platelet-activating factor that enhances the action of other agonists (such as ADP and thrombin 1) and is involved in broader inflammatory processes, leading to major thrombo-inflammatory disorders and diseases [61,62].

### 3.6. ATR-FTIR Analysis of Amphiphilic Extracts from WKGs and WKB

The main peaks in the ATR-FTIR spectra of water kefir grains appear at 3901 cm^−1^, 3334 cm^−1^, 2969 cm^−1^, 2354 cm^−1^, 2093 cm^−1^, 1667 cm^−1^, 1376 cm^−1^, 1119 cm^−1^, 948 cm^−1^, 813.4 cm^−1^ and 653.7 cm^−1^. In the spectrum of water kefir beverage, the main peaks are observed at 3893 cm^−1^, 3331 cm^−1^, 2969 cm^−1^, 2355 cm^−1^, 2091 cm^−1^, 1664 cm^−1^, 1376 cm^−1^, 1120 cm^−1^, 948.4 cm^−1^, 814.2 cm^−1^ and 656 cm^−1^. As can be seen, the extract of water kefir grains had a similar ATR-FTIR spectroscopic profile to that obtained for the extract of water kefir beverage. The similarity of these spectra suggests the presence of similar characteristic groups in the compounds of the two samples.

Table 5 present the main peaks in the ATR-FTIR spectra of the WKGs-TAC and WKB-TAC extracts compared to those obtained for the five standards used (β-carotene, gallic acid, catechin, quercetin, and polar lipids). According to the obtained spectra of both the sample extracts and the standards, several characteristic peaks are identified that can facilitate the structural analysis of the bioactive substances present in the amphiphilic lipid extracts of water kefir grains and beverages [63]. More specifically, the broad peak observed at about 3330 cm^−1^ in the spectra of the samples indicates the presence of hydroxyl (-OH) groups and also appears in the spectra of gallic acid, catechin, and quercetin. The peak at 2969 cm^−1^ corresponds to the stretching vibrations of single C-H bonds and is also present in the spectra of β-carotene and polar lipids. The peak observed at about 1660 cm^−1^ (at 1667 cm^−1^ in the WKGs-TAC extract spectrum and at 1664 cm^−1^ in the WKB-TAC extract spectrum) is indicative of C=C double bonds and is consistent with that found in the spectra of polar lipids containing unsaturated fatty acids and carotenoids with double bonds. The three peaks appearing in the 1580–1300 cm^−1^ region are probably due to the stretching vibrations of the C-H and C=C-C bonds of the aromatic ring of phenolic components [64], confirming the presence of rings in the structure of the compounds in the water kefir samples. In the fingerprint region, at about 1120 cm^−1^, the absorption peak of the ether bond (-C-O-C-) occurs, which is also found in the spectra of catechin and quercetin. The band appearing at about 950 cm^−1^ corresponds to hydrogen atoms bonded to sp2 hybridized carbon atoms (=C-H2) and is also found in the spectrum of β-carotene. Lastly, the peak appearing at about 810 cm^−1^ belongs to the region of out-of-plane C-H bending vibrations (900–690 cm^−1^), which are characteristic of aromatic rings. In the same region, peaks are also found in the spectra of β-carotene, gallic acid, catechin, and quercetin. This comprehensive spectral analysis indicates the presence of unsaturated polar lipids (such as glycolipids and phospholipids), β-carotene, and bioactive flavonoids in the samples of the water kefir grains and beverages. Indeed, various products fermented from water kefir as well as from individual water kefir microorganisms have shown increased concentrations of phenolic compounds, including gallic acid, catechin, and quercetin [65,66]. At the same time, the presence of carotenoid pigments, such as β-carotene, in water kefir made from fruit has been suggested as a possible cause of the yellow color of the beverages [67]. In addition, various polar lipids have been detected in the cells of several microorganisms of water kefir, such as the bacterial genus *Lactobacillus* and the bacterium *Zymomonas mobilis* [68,69].

Interestingly, bioactive compounds were identified not only in the water kefir beverage extract but also in the extract of unactivated/uncultivated water kefir grains. Therefore, it is confirmed that the bioactivity of water kefir is not solely due to the bioactive metabolites produced by its microbiota during fermentation but also to the beneficial microorganisms themselves, as well as to the synergistic action of the above two [11]. In addition to the presence of such bioactive compounds in water kefir grains, sugars have also been suggested to be present, as their characteristic absorptions at 1500–900 cm^−1^ are observed in the corresponding spectrum [70]. These sugars are probably present in the cell wall structure of bacteria and yeasts of water kefir [71,72].

Spectra obtained from ATR-FTIR analysis of water kefir beverages in other studies show similarities with the spectrum of our beverage [73,74]. These similarities include the appearance of two peaks at approximately 3650–3000 cm^−1^ and 1600 cm^−1^, which are indicative of -OH bond vibrations in the molecules of water, which is the major component of the fermentation medium. Furthermore, the appearance of a peak near 2300 cm^−1^ is attributed to the presence of carbon dioxide (CO_2_), while in the 1500–900 cm^−1^ region, characteristic sugar absorptions appear, including those located at 1300–1000 cm^−1^ and 1470–1370 cm^−1^, which are associated with the bending of C-H groups. Of course, in our case, the use of apple pomace as a fermentation substrate should also be taken into account. The observation in our spectrum of some peaks similar to those appearing in the spectrum of apple pomace, including the broad peak in the 3400–3200 cm^−1^ region due to the presence of polyphenol groups, and the peak at 2969 cm^−1^ corresponding to the C-H tension of the aliphatic carbon chain, suggest the presence of apple pomace compounds in the final fermentation product (beverage) [75]. Examples of such apple pomace components are the bioflavonoids catechin and quercetin [56], as well as β-carotene [30], whose presence has been established in the fermented beverage.

However, the absence from the beverage spectrum of some peaks attributed to the apple pomace polysaccharides that appear in the spectrum of apple pomace, including peaks at 1046–1020 cm^−1^ that are due to the stretching vibrations of the C-C, C-OH, and C-H groups of cellulose and hemicellulose, as well as peaks at 1741–1735 cm^−1^ corresponding to the more prevalent group of pectin and hemicellulose (C=O), indicates that the sugars contained in the fermentation product are not mainly derived from apple pomace but from water kefir grains [76]. Indeed, ATR-FTIR analyses of activated/cultured water kefir grains have confirmed their polysaccharide character [77,78,79]. More specifically, in the obtained spectra, peaks were observed in the 3600–3000 cm^−1^ region attributed to the -OH groups of water and carbohydrates, at around the 3030–2800 cm^−1^ zone due to the vibrations of C-H, both for the double bonds in alkenes and/or aromatic rings, but also in the methyl and methylene groups, and in the 1200–900 cm^−1^ region attributed mainly to the vibrations of the ring and side groups of carbohydrates (-C-O-C-, C-OH, C-H). The peaks in the latter region could be due to the vibrations of glucose units, which constitute the dextran produced by water kefir grains [77].

### 3.7. Fatty Acid Composition of the Amphiphilic Extracts from WKGs and WKB

The fatty acid composition of PL in the TAC extracts of the samples was determined by liquid chromatography-mass spectrometry (LC−MS) after saponification of all TAC extracts, and the results obtained are presented in Table 6. Saturated fatty acids (SFA) were the most abundant in both extracts, followed by smaller amounts of monounsaturated fatty acids (MUFA) and less abundant polyunsaturated fatty acids (PUFA). Specifically, the most abundant SFAs in both the water kefir grains and the water kefir beverage were stearic acid (49.8% and 65.4%, respectively) and palmitic acid (24.9% and 25.3%, respectively), while the main MUFA was oleic acid (11.5% and 3.1%, respectively). In addition, PUFAs, such as omega-6 PUFA linoleic acid, omega-3 PUFA alpha linolenic acid (ALA), and EPA, were also detected in small but appreciable amounts in both extracts.

LC−MS analysis of TAC extracts of unactivated/uncultivated water kefir grains showed the presence of various saturated and unsaturated fatty acids in their polar lipids. Indeed, most of these fatty acids, including stearic acid, palmitic acid, oleic acid, palmitoleic acid, myristic acid, and lauric acid, have been detected as lipid components of the membranes of various bacteria and yeasts isolated from kefir [80,81,82]. Therefore, the identification of these fatty acids in the polar lipids of the fermentation product, as well as their bioactivity induced by them, is partly due to the presence of these microorganisms themselves in the fermented beverage.

In addition, various lactic bacterial strains have been reported to have the ability to synthesize fatty acids during the water kefir fermentation process [83,84]. Similar to our beverage, increased amounts of saturated and unsaturated fatty acids have also been observed in other non-dairy kefir products, with the main SFAs being palmitic and stearic acids, and the main UFAs being oleic, linoleic, and linolenic acids [85,86]. Furthermore, a higher average content of SFAs than UFAs has also been reported in other kefir fermentation products [87,88]. Some of the fatty acids detected in the water kefir beverage could have been derived from apple pomace used as a fermentation substrate, in which they were also detected. More specifically, examples of such fatty acids include both the SFAs stearic and palmitic acid, MUFA oleic acid, and omega-6 PUFA linoleic acid, which are abundant in apple by-products [89], and the omega-3 PUFAs ALA and EPA, which are found in small but appreciable amounts in apple pomace [17].

In previous studies, the bioactivity of polar lipid extracts of apple pomace, including their anti-inflammatory and antithrombotic capacities, was associated with their high content of UFAs, especially omega-3 and omega-6 PUFAs [5,17]. Interestingly, the observed *n*-6/*n*-3 PUFA ratio in the polar lipids of the apple pomace-based water kefir beverage (≈3/1) was significantly lower than that reported for unfermented apples (5/1) [90]. The above finding demonstrates the important role of the water kefir fermentation process in enhancing the bioactivity of the fermentation raw material, considering that the lower the *n*-6/*n*-3 PUFA ratio, the greater the prophylactic anti-inflammatory benefits against various chronic disorders related to inflammation and platelet aggregation, and vice versa [91].

The extracts of water kefir beverage revealed higher anti-inflammatory and antioxidant activity than grain extracts, but lower content of UFAs (and individually MUFAs and PUFAs). The above fact suggests that besides unsaturated fatty acids, other bioactive compounds contained in the polar lipid extracts of the samples contribute to their bioactivity, such as amphiphilic phenolic compounds, the presence of which is associated with the antioxidant and antiplatelet capacity of these extracts. Indeed, several bioflavonoids, such as catechin, quercetin, and resveratrol, as well as their metabolites produced after fermentation, demonstrate potent anti-inflammatory activity against PAF signaling pathways in synergy with polar lipids, with which they may coexist [6,8]. Nevertheless, the presence of detectable amounts of several fatty acids in our samples that have been reported for their potential antioxidant and anti-inflammatory activities, including SFA stearic acid [92,93], MUFAs oleic [94], and palmitoleic acid [95,96], as well as ω-3 and ω-6 PUFAs [97], potentially enhanced the bioactivity of our extracts.

LC−MS analysis also revealed the presence of free fatty acids in the polar lipid extracts of the water kefir grains and water kefir beverage (Table 7). Even in this form, most of the fatty acids were saturated, with smaller amounts of monounsaturated and polyunsaturated fatty acids. The most abundant SFAs were again stearic and palmitic acids, while the main MUFA was oleic acid in both extracts. Furthermore, the omega-3 polyunsaturated fatty acid EPA was found in small but detectable amounts in both samples. 

In previous studies, the detection of free fatty acids has also been reported in both milk kefir products [88] and water kefir products [85]. The formation of these free fatty acids in the fermentation product is attributed to the action of lipolytic enzymes produced by kefir microorganisms during fermentation [85]. Interestingly, more saturated fatty acids were released as free fatty acids by this process (Table 7), while more unsaturated fatty acids remained bound in the polar lipid bioactive (Table 6) as part of the overall structures of the PL bioctives in the TAC extracts, explaining their observed higher biofunctionality in comparison to the TLC extracts.

### 3.8. Structural Elucidation of the PL Biocatives Present in the Amphiphilic Extracts from WKGs and WKB

With respect to the LC−MS structural analysis of PL in the TAC extracts from both water kefir grains(WKGs) and apple pomace-based fermentation beverage (WKB), survey scans in the negative ion mode between 500 and 1000 *m*/*z* demonstrated that the main glycolipid class identified were sphingosine-based ones, specifically several molecular species of ceramides(Cer) and hexosylceramides (HexCer), while the main phospholipid classes identified were glycerol-based ones (GP, glycerolphospholipids), such as several molecular species of phosphatidylcholines (PC), phosphatidylethanolamines (PE), phosphatidylinositols (PI) and phosphoserines (PS) (Table 8). Moreover, in all PL classes, both diacyl PLs and alkyl-acyl PLs of either glycerol-based phospholipids (PC, PE, PI, PS) and sphingosine-based glycolipids were detected. Additionally, glycerolipids, mostly SQDG and DGDG, were identified in the WKB extracts. These results are consistent with those reported in previous studies [98,99,100,101,102], while for such amphiphilic polar lipid bioactives several biological effects have been reported against inflammation-related disorders [103,104,105,106,107].

Similar to what was observed by the quantification of the fatty acid composition of water kefir grains and beverage PLs, most of these PL bioactives were found to mainly contain the SFAs stearic (C18:0) and palmitic (C16:0), followed by MUFA oleic acid (C18:1 c9) and palmitoleic acid (C 16:1 c9), but also the PUFA linoleic, DHA and EPA, with the SFA tending to be present in the *sn*-1 position, while the UFAs (either MUFA or PUFA) were predominantly present at the *sn*-2 position of these PLs. This positional specificity significantly influences the biochemical properties and functions of PLs, including membrane fluidity and enzyme interactions [108], such as the aforementioned interaction with PLA2, which provides anti-inflammatory intracellular signaling [2,103].

In the primary classes of apple PLs, specifically PC and PE, both alkyl-acyl and di-acyl variants were identified, with MUFAs or PUFAs predominantly occupying the sn-2 position of their glycerol backbones. These phospholipids exhibit notable anti-inflammatory properties against PAF by acting as potent PAF antagonists or, in certain cases, as less active agonists. They exert their effects by inhibiting the binding of PAF to its specific receptor (PAF-r), thereby preventing PAF-induced activation of various cell types, including platelets, and disrupting the thrombo-inflammatory cascades associated with the PAF/PAF-r pathway [8,103,104,108]. In platelets, antagonistic or agonistic inhibition of PAF activity by bioactive phospholipids attenuates PAF-induced activation and aggregation. Consequently, these actions provide protective and therapeutic effects, contributing to the prevention of cardiovascular diseases and tumor metastasis [2,103].

Additionally, following digestion, some portion of these dietary PL bioactives can be integrated into the lipoproteins and cell membranes of several cells. This incorporation may positively modulate inflammatory responses by activating mechanisms that enhance HDL levels and functionality [104]. Moreover, these PL bioactives influence cellular lipid signaling pathways related to PAF activity, thereby providing protection against thrombo-inflammatory manifestations involved in numerous diseases [2,103].

Interestingly, bioactive glycolipids were detected in WKB extracts but were absent in WKGs extracts. The presence of these apple pomace glycolipids in the extracts of the beverage enhanced their anti-inflammatory and antiplatelet activities, as has also been reported by Tsoupras et al. [5]. Apart from that, glycolipids such as SQDG and DGDG exhibit notable anti-tumor properties. In general, these PL bioctives selectively inhibit cancer cell proliferation, halt the G1 phase cell cycle, induce apoptosis, and enhance tumor necrosis [105,106] through mechanisms including inhibiting angiogenesis and suppressing cell proliferation markers like KI-67, PCNA, and Cyclin E [107].

## 4. Conclusions

This is the first study demonstrating that unactivated water kefir grains and their apple pomace-based fermented beverage contain bioactive compounds with strong antioxidant capacity and, at the same time, potent antiplatelet activity against not only the mediator of inflammation and thrombosis PAF, but also the classic platelet agonist ADP. The lipid extracts of amphiphilic components of the water kefir grains and beverage showed the strongest antioxidant and anti-inflammatory/antithrombotic activity, which seems to be consistent with the higher total phenolic content (TPC) of these extracts compared to that found for the lipid extracts of the lipophilic components of the samples, which exhibited none or low bioactivity in the antioxidant and antiplatelet activity assays. Therefore, the most bioactive compounds are mainly present in the amphiphilic extracts of water kefir. This important result is related to the possible beneficial effects of water kefir on health, as the amphiphilic bioactive constituents of water kefir and its products can diffuse directly into the bloodstream after absorption from the intestine, acting through various antioxidant and antiplatelet mechanisms against oxidative stress and inflammation, thereby preventing the onset and development of related diseases.

In addition, the detection of bioactive components in the extracts of unactivated/uncultivated water kefir grains, such as phenolic compounds and carotenoids, as well as different types of free and esterified saturated and unsaturated fatty acids that were identified by LC−MS analysis, and the strong antioxidant and antiplatelet activity observed in the amphiphilic extracts of the grains confirms the contribution of the water kefir microorganisms themselves to the bio-functionality of the fermented product. At the same time, the extracts of the water kefir beverage showed higher antioxidant and anti-inflammatory/antithrombotic activities than the grain extracts, to which the presence of bioactive metabolites produced by kefir microflora and/or biochemical alteration of apple pomace-derived bioactive components during fermentation seems to have contributed. Interestingly, the water kefir beverage based on apple pomace showed a lower *n*-6/*n*-3 PUFA ratio and a stronger inhibitory activity against the signaling pathways of platelet agonists PAF and ADP than has been reported for unfermented apple pomace in the literature. The above finding indicates the stronger anti-inflammatory and antithrombotic activity of the water kefir-fermented apple pomace than that of the unfermented apple pomace, confirming the important role of the fermentation process in enhancing the bioactivity of the raw material.

The overall findings of the present study highlight the potential application of water kefir grains not only for the production of bioactive water kefir beverages but also as a source of bioactive metabolites, such as phenolic compounds, carotenoids, and polar lipids, which could be used as novel constituents for the development of various health-promoting bio-functional products acting against oxidative stress and inflammation-related disorders. In addition, through the aforementioned results, it is proposed that biowastes, such as apple pomace, can be utilized as fermentation substrates for the production of water kefir, which could contribute to the development of environmentally friendly, innovative health-promoting bio-functional products applicable to the food, cosmetic, and even pharmaceutical industries in a circular economy context.

## Figures and Tables

**Table 1:** Yield of extracted TAC extracts from WKGs and WKB expressed as g/100 g grains or 100 mL beverage.

	Yield of Extraction		
	g/100 g Grains or 100 mL Beverage		
Extract	Minimum	Median	Maximum
WKGs-TAC	0.160	0.410	0.560
WKGs-TLC	0.064	0.088	0.187
WKB-TAC	0.002	0.020	0.048
WKB-TLC	0.002	0.003	0.004

Abbreviations: WKGs-TAC, amphiphilic lipids extracted from water kefir grains; WKGs-TLC, lipophilic lipids extracted from water kefir grains; WKB-TAC, amphiphilic lipids extracted from water kefir beverage; WKB-TLC, lipophilic lipids extracted from water kefir beverage.

**Table 2:** Total carotenoid content of extracts from WKGs and WKB.

	Total Carotenoid Content (TCC)		
	mg CE/g Extract		
Extract	Minimum	Median	Maximum
WKGs-TAC	10.14	11.71	28.50
WKGs-TLC	15.61	19.09	50.63
WKB-TAC	19.37	114.00	192.00
WKB-TLC	108.57	122.50	315.56

Abbreviations: WKGs-TAC, amphiphilic lipids extracted from water kefir grains; WKGs-TLC, lipophilic lipids extracted from water kefir grains; WKB-TAC, amphiphilic lipids extracted from water kefir beverage; WKB-TLC, lipophilic lipids extracted from water kefir beverage.

**Table 3:** Total phenolic content of extracts from WKGs and WKB.

	Total Phenolic Content (TPC)		
	mg GAE/g Extract		
Extract	Minimum	Median	Maximum
WKGs-TAC	92.26	101.63	245.54
WKGs-TLC	3.72	129.87	160.43
WKB-TAC	47.62	351.19	1404.76
WKB-TLC	370.37	386.90	442.18

Abbreviations: WKGs-TAC, amphiphilic lipids extracted from water kefir grains; WKGs-TLC, lipophilic lipids extracted from water kefir grains; WKB-TAC, amphiphilic lipids extracted from water kefir beverage; WKB-TLC, lipophilic lipids extracted from water kefir beverage.

**Table 4:** Antioxidant activities of the extracts of WKGs and WKB.

	ABTS			TEAC			FRAP		
	μmol TE/g Extract								
Extract	Minimum	Median	Maximum	Minimum	Median	Maximum	Minimum	Median	Maximum
WKGs-TAC	5.12	8.62	10.06	0.0005	0.0023	0.0040	21.65	21.91	53.69
WKGs-TLC	ND	ND	ND	ND	ND	ND	0.89	4.12	6.16
WKB-TAC	5.84	44.62	199.76	0.0028	0.0083	0.0507	8.26	46.02	351.01
WKB-TLC	ND	ND	ND	ND	ND	ND	14.27	14.27	15.85

Abbreviations: WKGs-TAC, amphiphilic lipids extracted from water kefir grains; WKGs-TLC, lipophilic lipids extracted from water kefir grains; WKB-TAC, amphiphilic lipids extracted from water kefir beverage; WKB-TLC, lipophilic lipids extracted from water kefir beverage; ND, non-detected.

**Table 5:** Characteristic infrared (IR) absorption peaks for TAC extracts from WKGs and WKB, with corresponding functional group assignments.

Peak (cm^−1^)	Extract		Bond/Functional Group Correlation
	WKGs-TAC	WKB-TAC	
Broad peak at about 3330 cm^−1^	+	+	O-H (hydroxyl) bonds, characteristic of this functional group in phenolic compounds (e.g., gallic acid, catechin, and quercetin)
Peaks at about 3000 cm^−1^	+	+	Vibrations of C-H, both for the double bonds in alkenes and/or aromatic rings but also in single -C-H (alkyl) bonds, also present in β-carotene and polar lipids
Peak at about 1660 cm^−1^	+	+	C=C bonds, also present in polar lipids containing unsaturated fatty acids and in carotenoids with double bonds)
Three peaks in the 1580–1300 cm^−1^ region	+	+	Stretching vibrations of C-H and C=C-C bonds of the aromatic ring of phenolic compounds
Peak at about 1120 cm^−1^	+	+	C-O-C (ether) bond, also present in catechin and quercetin
Peak at about 950 cm^−1^	+	+	Hydrogen atoms bonded to sp2 hybridized carbon atoms (=C-H2 bond), also found in β-carotene
Peak at about 810 cm^−1^	+	+	Out-of-plane C-H bending vibrations (900–690 cm^−1^), characteristic of aromatic rings that are also found in β-carotene, gallic acid, catechin, and quercetin

**Table 6:** The fatty acid profile of the saponified polar lipids of the TAC extracts from WKGs and WKB, expressed for each FA as a percentage composition of the total fatty acids in each sample assessed (mean ± standard deviation (SD); *n* = 3).

Fatty Acid Emperical Name	Lipid Number	WKGs-TAC	WKB-TAC
Caprylic	C8:0	0.063 ± 0.002	0.134 ± 0.016
Pelargonic	C9:0	0.229 ± 0.009	0.465 ± 0.010
Capric	C10:0	ND	ND
Lauric	C12:0	0.139 ± 0.013	0.424 ± 0.035
Tridecylic	C13:0	ND	ND
Myristic	C14:0	0.558 ± 0.063	0.826 ± 0.036
Pentadecylic	C15:0	ND	ND
Palmitic	C16:0	24.876 ± 1.601	25.293 ± 4.178
Palmitoleic	C16:1 c9 (n7 MUFA)	9.305 ± 0.272	0.789 ± 0.118
Margaric	C17:0	1.156 ± 0.134	2.388 ± 0.598
Stearic	C18:0	49.801 ± 1.731	65.423 ± 4.952
Oleic	C18:1 c9 (n9 MUFA)	11.517 ± 0.255	3.135 ± 0.361
Linoleic	C18:2 c9,12 (n6 PUFA)	0.523 ± 0.063	0.870 ± 0.048
Linolenic (α + γ)	C18:3 c9,12,15 (n3 PUFA)	0.099 ± 0.010	0.133 ± 0.008
Stearidonic	C18:4 c6,9,12,15 (n3 PUFA)	ND	ND
Nonadecylic	C19:0	ND	ND
Arachidic	C20:0	ND	ND
Gadoleic	C20:1 c9 (n11 MUFA)	0.614 ± 0.012	ND
DihomoLinoleic	C18:2 c10,12 (n6 PUFA)	0.116 ± 0.013	ND
Dihomolinolenic	C20:3 c8,11,14 (n6 PUFA)	ND	ND
Arachidonic	C20:4 c5,8,11,14 (n6 PUFA)	0.140 ± 0.004	ND
EPA	C20:5 c5,8,11,14,17 (n3 PUFA)	0.316 ± 0.066	0.121 ± 0.013
Docosadienoic	C22:2 c13,16 (n6 PUFA)	ND	ND
Eranthic	C22:3 c5,13,16 (n6 PUFA)	ND	ND
Ardenic	C22:4 c7,10,13,16 (n6 PUFA)	ND	ND
DPA	C22:5 c7,10,13,16,19 (n3 PUFA)	ND	ND
DHA	C22:6 c4,7,10,13,16,19 (n3 PUFA)	0.548 ± 0.095	ND
SFA		76.822 * ± 0.328	94.953 * ± 0.498
UFA		23.178 ± 0.328	5.047 ± 0.498
MUFA		21.436 ** ± 0.333	3.924 ** ± 0.448
PUFA		1.742 ± 0.239	1.123 ± 0.053
n3PUFA		0.963 ± 0.172	0.253 ± 0.021
n6PUFA		0.779 ± 0.073	0.870 ± 0.048
n6/n3		0.820 ± 0.095	3.451 ± 0.347

* denotes a statistically significant difference (*p* < 0.05) between SFA and UFA. ** denotes a statistically significant difference (*p* < 0.05) between MUFA and PUFA. Abbreviations: *n*-3, omega-3 PUFA; *n*-6, omega-6 PUFA; PUFAs, polyunsaturated fatty acids; MUFAs, monounsaturated fatty acids; SFAs, saturated fatty acids; ALA, alpha linolenic acid; LA, linoleic acid; EPA, eicosapentaenoic acid; DPA, docosapentaenoic acid; DHA, docosahexaenoic acid; ND, non-detectable (defined as fatty acids detected with lower than 0.005% contribution to the overall fatty acid content).

**Table 7:** The free fatty acid profile of the non-saponified TAC extracts from WKGs and WKB, expressed for each FA as a percentage composition of the total fatty acids in each sample assessed (mean ± standard deviation (SD); *n* = 3).

Fatty Acid Emperical Name	Lipid Number	WKGs-TAC	WKB-TAC
Caprylic	C8:0	ND	ND
Pelargonic	C9:0	0.468 ± 0.025	0.458 ± 0.028
Lauric	C12:0	ND	ND
Tridecylic	C13:0	ND	ND
Myristic	C14:0	0.714 ± 0.055	ND
Pentadecylic	C15:0	0.680 ± 0.191	ND
Palmitic	C16:0	24.943 ± 1.361	25.544 ± 1.781
Palmitoleic	C16:1 c9 (n7 MUFA)	5.056 ± 0.091	ND
Margaric	C17:0	2.862 ± 0.059	ND
Stearic	C18:0	53.091 ± 1.242	66.794 ± 1.831
Oleic	C18:1 c9 (n9 MUFA)	9.569 ± 1.289	7.051 ± 0.156
Linoleic	C18:2 c9,12 (n6 PUFA)	1.673 ± 0.079	ND
Linolenic (α + γ)	C18:3 c9,12,15 (n3 PUFA)	0.200 ± 0.023	ND
Stearidonic	C18:4 c6,9,12,15 (n3 PUFA)	ND	ND
Nonadecylic	C19:0	ND	ND
Gadoleic	C20:1 c9 (n11 MUFA)	ND	ND
DihomoLinoleic	C18:2 c10,12 (n6 PUFA)	ND	ND
Dihomolinolenic	C20:3 c8,11,14 (n6 PUFA)	ND	ND
Arachidonic	C20:4 c5,8,11,14 (n6 PUFA)	ND	ND
EPA	C20:5 c5,8,11,14,17 (n3 PUFA)	0.345 ± 0.036	0.153 ± 0.023
Docosadienoic	C22:2 c13,16 (n6 PUFA)	ND	ND
Eranthic	C22:3 c5,13,16 (n6 PUFA)	ND	ND
Ardenic	C22:4 c7,10,13,16 (n6 PUFA)	ND	ND
DPA	C22:5 c7,10,13,16,19 (n3 PUFA)	ND	ND
DHA	C22:6 c4,7,10,13,16,19 (n3 PUFA)	0.399 ± 0.015	ND
SFA		82.758 * ± 1.328	92.796 * ± 0.155
UFA		17.242 ± 1.328	7.204 ± 0.155
MUFA		14.625 ** ± 1.353	7.051 ** ± 0.156
PUFA		2.617 ± 0.146	0.153 ± 0.023
n3PUFA		0.944 ± 0.071	0.153 ± 0.023
n6PUFA		1.673 ± 0.079	-
n6/n3		1.775 ± 0.068	-

* denotes a statistically significant difference (*p* < 0.05) of SFA compared to UFA. ** denotes a statistically significant difference (*p*< 0.05) of MUFA compared to PUFA. Abbreviations: *n*-3, omega-3 PUFA; *n*-6, omega-6 PUFA; PUFAs, polyunsaturated fatty acids; MUFAs, monounsaturated fatty acids; SFAs, saturated fatty acids; ALA, alpha linolenic acid; LA, linoleic acid; EPA, eicosapentaenoic acid; DPA, docosapentaenoic acid; DHA, docosahexaenoic acid; ND, non-detectable (defined as fatty acids detected with lower than 0.005% contribution to the overall fatty acid content).

**Table 8:** Representative molecular species of the main classes of polar lipid bioactive compounds detected in TAC extracts of water kefir grains and beverages by LC−MS analysis.

	TAC Extracts from WKG				TAC Extracts from WKB			
Main Classes of PLs	Elution Time (min)	Mr	Representative Molecular Species	Proposed Structures	Elution Time (min)	Mr	Representative Molecular Species	Proposed Structures
PC	10.2–10.3	792.9	PC O-38:6;O	(i.e PC O-16:0/22:6 or PC O-18:1/20:5)	10.2–10.3	734.4	PC 32:0;O	(i.e PC 18:0/14:0;O or 16:0/16:0;O)
	11.7–11.8	770.9	PC 36:2	(i.e PC 18:0/18:2)	11.7–11.8	742.6	PC 34:2	(i.e PC 16:0/18:2)
					10.2–10.3	744.4	PC 34:1	(i.e PC 18:0/16:1 or PC 16:0/18:1)
PE	9.0–9.2	792.9	PE O-40:6;O	(i.e PE O-18:0/22:6;O)	10.2–10.3	714.4	PE 34:2	(i.e PE 16:0/18:2)
	10.2–10.3	714.8	PE 34:2	(i.e PE 16:0/18:2)	10.2–10.3	698.4	PE O-34:3	(i.e PE 16:0/18:3)
					10.2–10.3	744.4	PE 36:1	(i.e PE 18:0/18:1)
					10.2–10.3	734.4	PE 34:0;O	(i.e., PE 18:0/16:0;O)
					11.7–11.8	742.6	PE 36:2	(i.e PE 18:0/18:2)
PI	9.9–10.1	835.5	PI 34:1	(i.e PI 18:0/16:1 or PI 16:0/18:1)	12.6–12.8	821.5	PI O-34:1	(i.ePI O-16:0/18:1)
	12.0–12.5	835.5	PI O-34:2;O	(i.e PI O-16:0/18:2)				
	12.6–12.8	821.5	PI O-34:1	(i.ePI O-18:0/16:1 or PI O-16:0/18:1)				
PS	9.9–10.1	792.9	PS O-36:0;O	(i.e., PS O-18:0/18:0;O)	10.2–10.3	734.4	PS 32:0	(i.e PS 18:0/14:0 or 16:0/16:0)
	9.9–10.1	808.8	PS O-38:6;O	(i.e PS O-16:0/22:6;O or PS O-18:1/20:5;O)	11.7–11.8	742.6	PS O-34:3	(i.e PS 16:0/18:3)
					10.2–10.3	744.4	PS O-34:2	(i.e PS 16:0/18:2)
Cer	12.1–12.5	520.9	Cer 34:1;O	(i.e., Cer18:0/16:1 or Cer 16:0/18:1)	12.1–12.5	520.9	Cer 34:1;O	(i.eCer18:0/16:1 or Cer 16:0/18:1)
	2.6–2.7	612.3	Cer 36:1;O5	(i.e., Cer 18:0/18:1;O5)	12.1–12.5	626.4	Cer 36:2;O6	(i.eCer 18:0/18:2;O6)
	12.6–12.8	626.4	Cer 36:2;O6	(i.eCer 18:0/18:2;O6)				
HexCer	10.2–10.3	792.9	HexCer 36:0;O6	(i.e., HexCer 18:0/18:0;O6)	10.2–10.3	792.9	HexCer 36:0;O6	(i.e., HexCer 18:0/18:0;O6)
	9.9–10.1	808.8	HexCer 38:6;O6	(i.eHexCer 16:0/22:6;O6 or HexCer 18:1/20:5;O6)	11.7–11.8	742.6	HexCer 36:1;O3	(i.eHexCer 18:0/18:1;O3)
SQDG					12.6–12.8	821.5	SQDG 34:0	(i.e., SQDG 18:0/16:0)
DGDG					13.0–13.1	915.6	DGDG 34:2	(i.e DGDG 16:0/18:2)

Abbreviations: PLs = polar lipids; PC = phosphatidylcholine; PE = phosphatidylethanolamine; PI = phosphoinositol; PS = phosphatidylserine; Cer = ceramides; HexCer = sphingosine-based hexosylceramides (glycolipids); SQDG = sulfoquinovosyldiacylglycerols; DGDG = digalactosyldiacylglycerols.

## Data Availability

The raw data supporting the conclusions of this article will be made available by the PI of the project and the corresponding author (A.T.) upon request.

## Supplementary Materials

The following supporting information can be downloaded at: https://www.mdpi.com/article/10.3390/antiox14020164/s1. Supplementary Figures S1–S3: Representative FTIR spectra, Fatty Acid Profile by LC−MS analysis, and a chromatogram of water kefir extract with some ESI-MS analysis, as observed and obtained during the analysis.
