# Supplementary material for: Antioxidant, Antithrombotic and Anti-Inflammatory Properties of Amphiphilic Bioactives from Water Kefir Grains and Its Apple Pomace-Based Fermented Beverage
Source: Antioxidants (Basel). 2025 Jan 29;14(2):164. doi: 10.3390/antiox14020164 (PMC11851739; doi:10.3390/antiox14020164)
Supplement: Supplementary file 1 [file antioxidants-14-00164-s001.zip › antioxidants-3417588-supplementary.pdf]

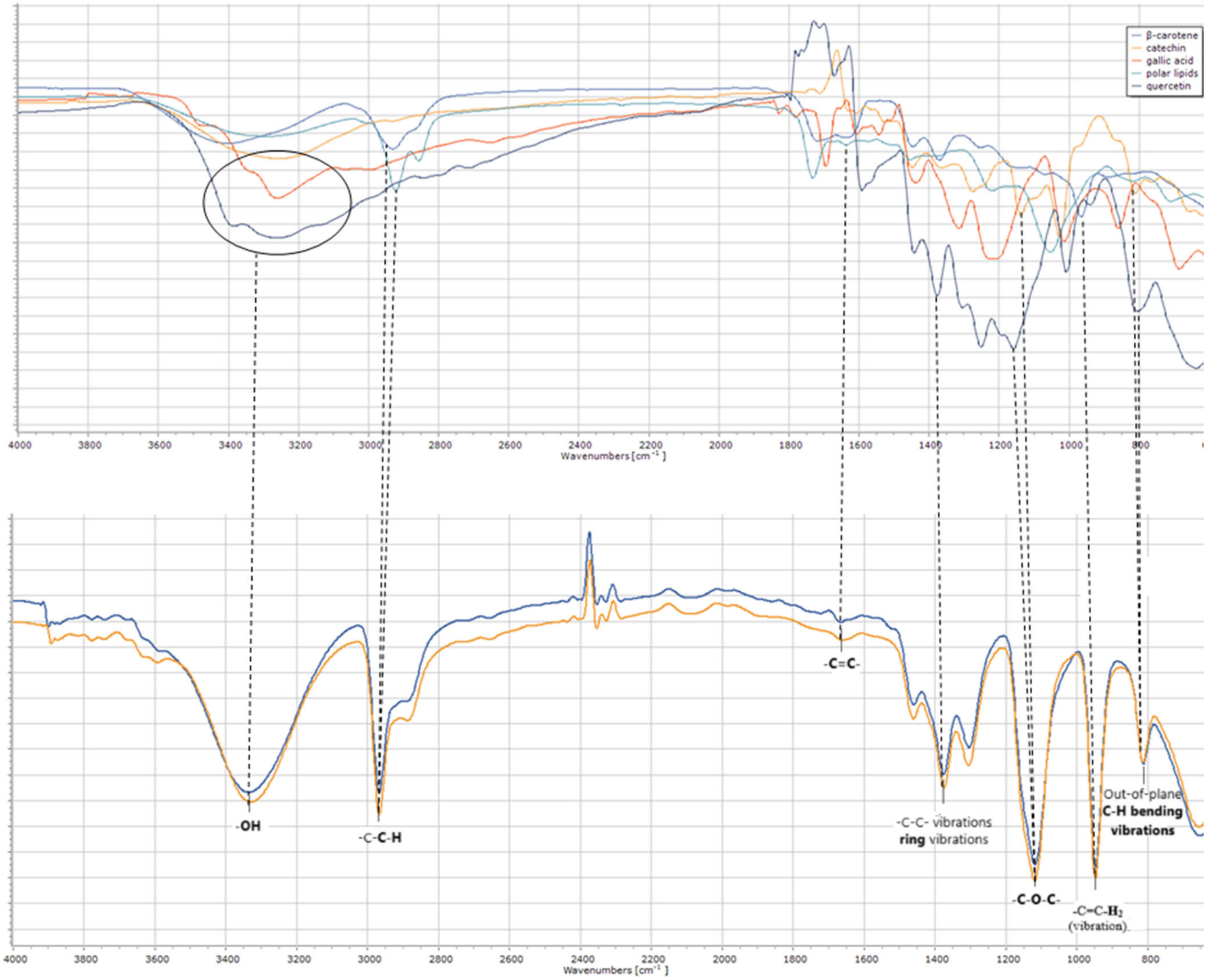

Supplementary Figure 1. ATR-FTIR spectra of the TAC lipid extracts of WKGs and WKB compared to those of the five standards. Abbreviations: WKGs-TAC, amphiphilic lipids extracted from water kefir grains; WKB-TAC, amphiphilic lipids extracted from water kefir beverage.

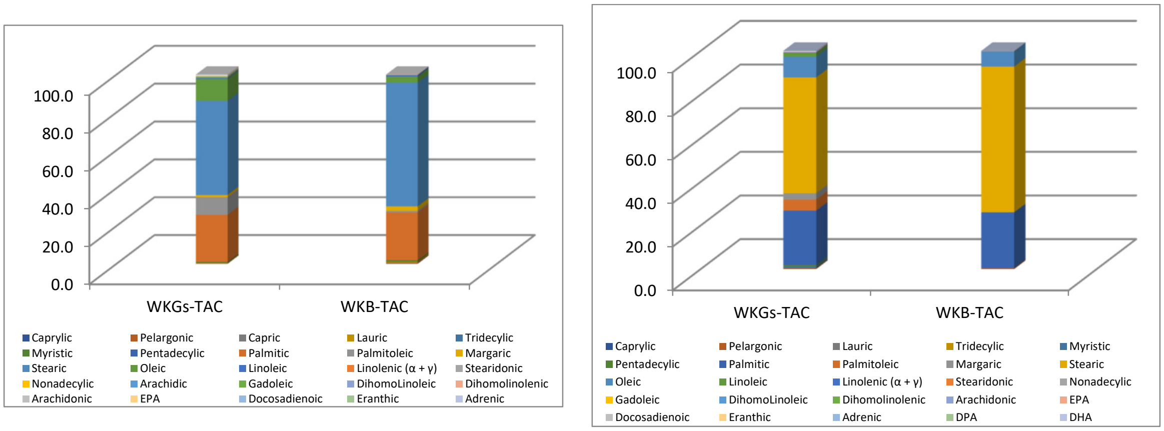

(**a**) (**b**)

Supplementary Figure 2. The saponified (**a**) and free (**b**) fatty acid profile of the TAC extracts from WKGs and WKB. Abbreviations: EPA, eicosapentaenoic acid; DPA, docosapentaenoic acid; DHA, docosahexaenoic acid.

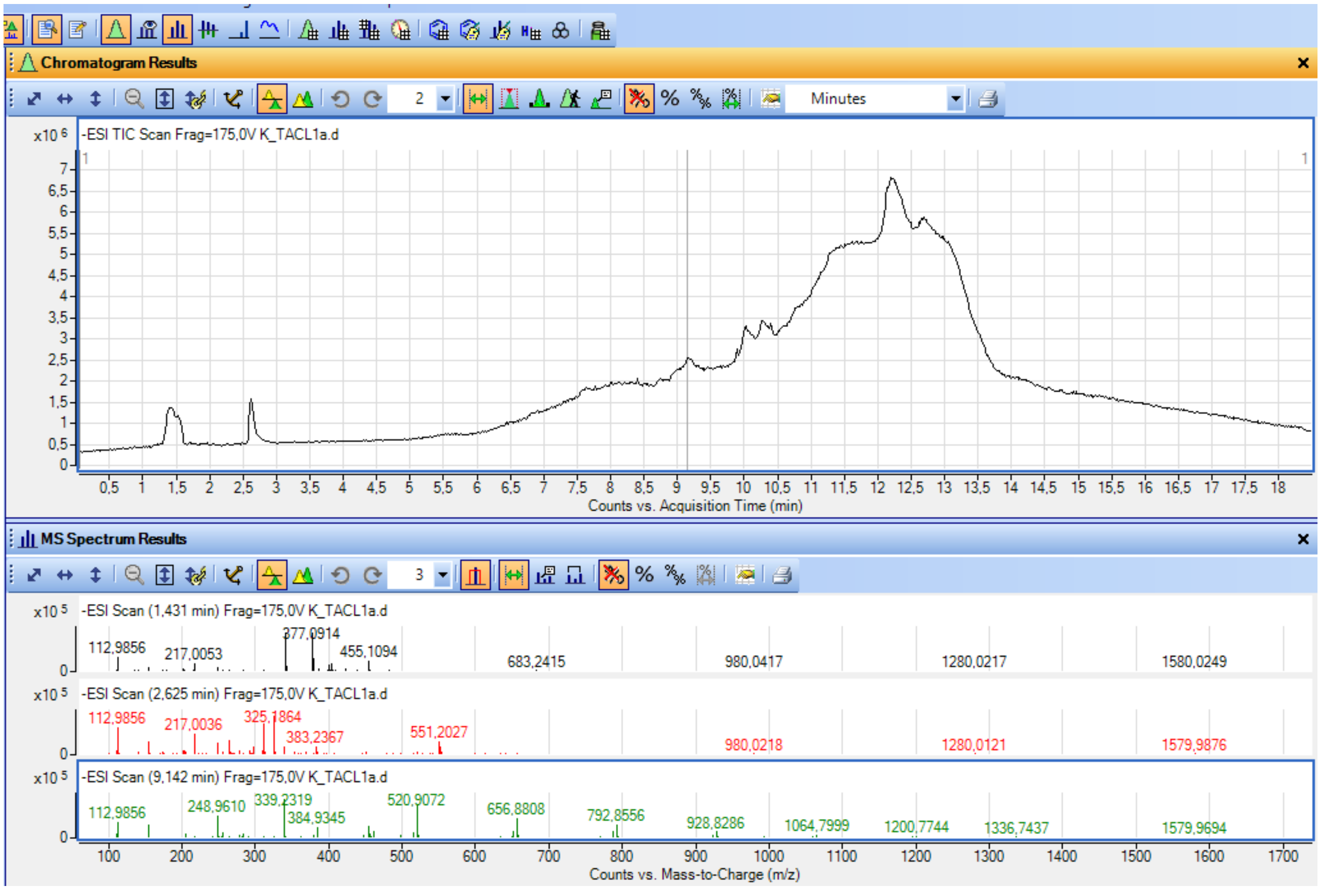

Supplementary Figure 3: Representative Chromatogram of water kefir TAC extract with some ESI-MS analysis, as observed and obtained during the Analysis
